# Identification of Potential Biomarkers and Small Molecule Drugs for Bisphosphonate-Related Osteonecrosis of the Jaw (BRONJ): An Integrated Bioinformatics Study Using Big Data

**DOI:** 10.3390/ijms24108635

**Published:** 2023-05-11

**Authors:** Kumarendran Balachandran, Roszalina Ramli, Saiful Anuar Karsani, Mariati Abdul Rahman

**Affiliations:** 1 Department of Craniofacial Diagnostics and Biosciences, Faculty of Dentistry, University Kebangsaan Malaysia, Jalan Raja Muda Abdul Aziz, Kuala Lumpur 50300, Malaysia; 2 Department of Oral and Maxillofacial Surgery, Faculty of Dentistry, Universiti Kebangsaan Malaysia, Kuala Lumpur 50300, Malaysia; 3 Institute of Biological Sciences, Faculty of Science, Universiti Malaya, Kuala Lumpur 50603, Malaysia

**Keywords:** bisphosphonate-related osteonecrosis of the jaw, bioinformatics, gene expression, molecular target

## Abstract

This study aimed to identify potential molecular mechanisms and therapeutic targets for bisphosphonate-related osteonecrosis of the jaw (BRONJ), a rare but serious side effect of bisphosphonate therapy. This study analyzed a microarray dataset (GSE7116) of multiple myeloma patients with BRONJ *(n* = 11) and controls (*n* = 10), and performed gene ontology, a pathway enrichment analysis, and a protein–protein interaction network analysis. A total of 1481 differentially expressed genes were identified, including 381 upregulated and 1100 downregulated genes, with enriched functions and pathways related to apoptosis, RNA splicing, signaling pathways, and lipid metabolism. Seven hub genes (*FN1, TNF, JUN, STAT3, ACTB, GAPDH,* and *PTPRC)* were also identified using the cytoHubba plugin in Cytoscape. This study further screened small-molecule drugs using CMap and verified the results using molecular docking methods. This study identified 3-(5-(4-(Cyclopentyloxy)-2-hydroxybenzoyl)-2-((3-hydroxybenzo[d]isoxazol-6-yl) methoxy) phenyl) propanoic acid as a potential drug treatment and prognostic marker for BRONJ. The findings of this study provide reliable molecular insight for biomarker validation and potential drug development for the screening, diagnosis, and treatment of BRONJ. Further research is needed to validate these findings and develop an effective biomarker for BRONJ.

## 1. Introduction

Bisphosphonate-related osteonecrosis of the jaw (BRONJ) is characterized by an exposed refractory bone in the area of the jaw, caused by an adverse effect of bisphosphonate (BP) treatment [1]. Currently, there are no effective biomarkers for BRONJ. The understanding of BRONJ pathophysiology remains theoretical. It is believed that aberrations in bone metabolism are caused by the deposition of bisphosphonates (BP) in the bone cause the homeostatic imbalance of osteoblasts and osteoclasts [2]. However, many studies have suggested a genetic link of the condition as not everyone treated with BP develops BRONJ [3,4].

Diagnostic bioinformatics is the application of bioinformatic techniques towards the development and improvement of diagnostic tests [5]. The impact of bioinformatics has been significant, as it allows for the efficient and accurate identification of diseases and the development of personalized treatment plans [6,7]. For example, cancer diagnostics using a large amount of genetic and molecular data has been used to identify genetic markers for different types of cancers [8,9]. Additionally, non-invasive prenatal testing (NIPT) has allowed for detection of chromosome abnormalities in fetal DNA [10]. In analyzing large data, bioinformatics has aided in the identification of new drug targets and the optimization of drug designs for the development of personalized medicine [11,12].

A broad spectrum of gene expression data is available online. If thoroughly investigated, these data may provide predictive insights into the molecular mechanisms underlying diseases. As such, this study aimed to utilize these data, together with powerful informatic tools, to unravel and identify potential biomarkers for BRONJ diagnosis, and to identify potential drugs and drug targets for the treatment of BRONJ.

A robust analytical method was used in this study. While an analysis of a single gene chip, as performed in this study, may yield false-positive results, we used a robust multichip array average (RMA) method, which integrated four important features: strong robustness to noise, incomplete ranking, significant scores in the result ranking, and high computational ranking. To the best of our knowledge, no previous study has used the RMA method that facilitated this study to identify differentially expressed genes (DEG) in BRONJ. A group from Korea used similar datasets for a combined predictor analysis and did not conduct any pre-processing of the data [13]. Using a non-parametric statistical analysis, they found 200 significant genes [13]. A more recent study used GSE7116 for data mining for anti-cancer therapeutics using the DGIdb database [14]. The GEO2R tool used in the previous anti-cancer therapeutic study poses several limitation [14,15]. GEO2R uses statistical tests to identify differentially expressed genes; however, these tests make certain assumptions about the data, such as the normality and homogeneity of variance, without the ability to pre-process the data. We have provided a summary of studies that have previously used GSE7116 in Table 1.

In this study, we performed gene ontology (GO), Kyoto Encyclopedia of Genes and Genomes (KEGG) enrichment, and protein–protein network analyses. We also provided the relationship between different genes and their regulatory networks, using the cytoHubba plugin of Cytoscape. Previous studies have successfully utilized this approach for biomarker discovery [20,21,22]. Furthermore, from the results of the above analysis, we sourced small drug molecules that interacted with key hub genes. To validate this, we used a molecular docking method to confirm the possible mechanism of action. The steps of this procedure are explained in our Section 4.1.

## 2. Results

### 2.1. Identification of DEGs in BRONJ

**Figure 1:**
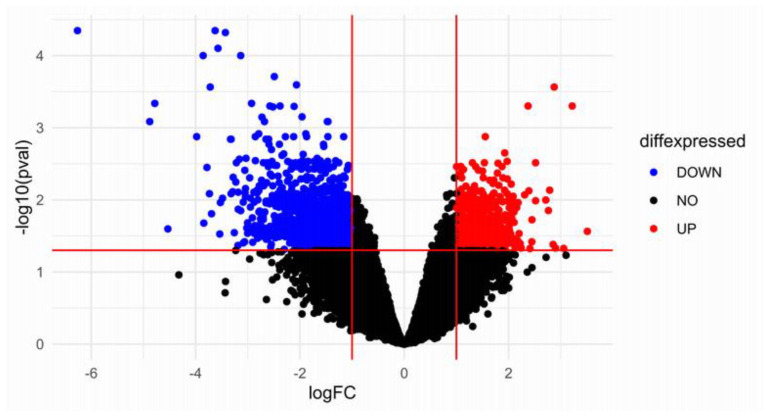
Identification of DEGs data from GEO dataset GSE7116. Volcano plot representing differential gene expression analysis between condition A and condition B. Genes that are significantly up-regulated (log2 fold change >1, adjusted *p*-value <0.05) are shown in red, while genes that are significantly down-regulated (log2 fold change < −1, adjusted *p*-value < 0.05) are shown in blue. The *x*-axis represents the log2 fold change in gene expression between BRONJ vs. control, while the *y*-axis represents the negative logarithm of the adjusted *p*-value (significance level) for each gene. Genes with high significance and large fold changes are located towards the top and sides of the plot, respectively.

Our search strategy yielded several relevant studies, and we downloaded and analyzed the microarray dataset GSE7116. This study was a platform based on GPL570, containing total samples from 11 MM patients with BRONJ and 10 MM patients without BRONJ. A volcano plot of the microarray results after RMA normalization (*p* < 0.05) is shown in Figure 1. A total of 1481 DEGs (381 upregulated and 1100 downregulated) were identified.

### 2.2. Functional and Network Analysis of DEGs

**Figure 2:**
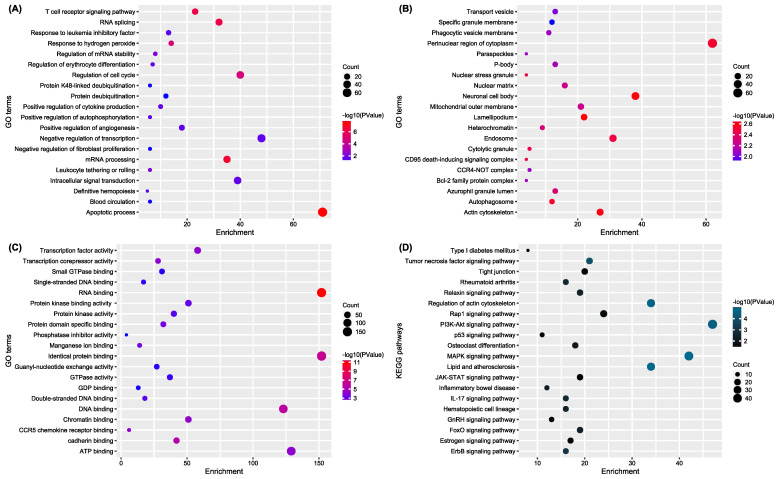
GO and KEGG analysis of the DEGs according to (**A**) biological process, (**B**) cellular components, (**C**) molecular functions, and (**D**) KEGG.

We used the Database for Annotation, Visualization, and Integrated Discovery (DAVID) to perform a functional analysis of these DEGs. As shown in Figure 2, we found that DEGs between the control and BRONJ groups were significantly enriched in the apoptotic process, T cell receptor signaling pathway, RNA splicing, RNA binding, PI3k-Akt signaling pathway, regulation of actin cytoskeleton, MAPK signaling, and lipid and atherosclerosis pathways.

### 2.3. PPI Network and Hub Genes

**Figure 3:**
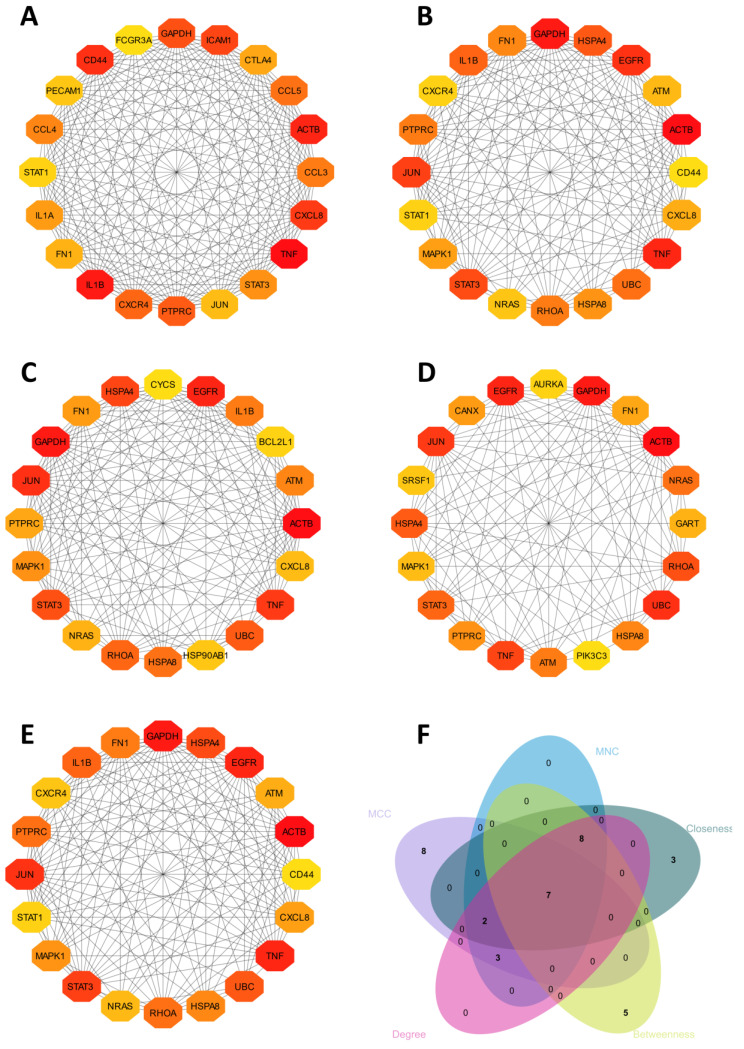
Displays a network analysis of the top 150 proteins based on their network parameters: (**A**) MCC, (**B**) MNC, (**C**) Closeness, (**D**) Betweenness, and (**E**) Degree, as well as a Venn diagram for hub genes. The nodes in the network represent proteins, and the edges represent their interactions. Panel (**F**) shows a Venn diagram of the hub genes, with the number of proteins in each intersection indicated. This figure provides insight into the identification of key proteins that may be involved in the network’s function and highlights their importance based on different network parameters.

**Figure 4:**
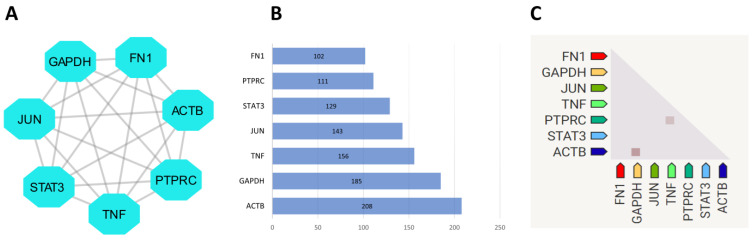
Displays a network analysis of hub genes, their degree, and co-expression. (**A**) shows the hub genes in the network. The nodes in the network represent genes/proteins, and the edges represent their interactions. (**B**) shows a bar chart of the degree of hub genes, which is the number of nodes and edges they interact with. The *x*-axis shows the number of interactions, and the *y*-axis shows the hub genes. (**C**) shows the co-expression of hub genes, which is the level of similarity in expression patterns between genes. This figure provides insight into the key genes that may be involved in the network’s function, their degree of interaction, and their co-expression patterns.

We used STRING to examine and identify possible PPI networks. A PPI network was constructed using the identified DEGs, which is presented in Figure 3. The cytoHubba plug-in in Cytoscape was used to determine the most significant hub gene(s) from the PPI network. We expanded the Venn diagram (Figure 3F) to intersect the most significant genes according to the density of the maximum neighborhood component (MNC), maximal clique centrality (MCC), density of maximum neighborhood component (DMNC), closeness, betweenness, and degree. This led to the identification of seven hub genes (Figure 4). Functional analyses of the seven hub genes were performed using DAVID. The expression patterns of these genes are provided in the Supplementary Materials.

### 2.4. Small Molecule Drug Screening

**Figure 5:**
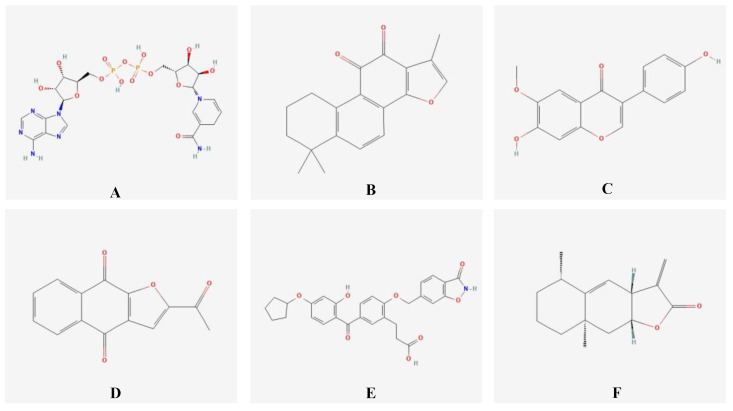
Chemical structures of six compounds: (**A**) 1,4-dihydronicotinamide adenine dinucleotide, (**B**) Tanshinone IIA, (**C**) Glycitein, (**D**) Napa-bucasin, (**E**) 3-(5-(4-(Cyclopentyloxy)-2-hydroxybenzoyl)-2-((3-hydroxybenzo[d]isoxazol-6-yl) methoxy) phenyl) propanoic acid, and (**F**) Alantolactone. The stereochemistry of the compounds is shown using wedges and dashes to indicate the three-dimensional arrangement of atoms in space. Chiral centers are indicated by R or S configurations, and the stereochemistry of specific positions in the molecule is indicated by dashed or solid wedges. The figure also highlights the active site of each molecule, which is an important feature for its function, and this can vary depending on the specific molecule and its interactions with other molecules.

We utilized the CMap network to analyze the seven identified DEGs. The top six compounds with the highest negative enrichment scores were identified as potential therapeutic targets for BRONJ, Alantolactone, 1,4-dihydronicotinamide adenine dinucleotide, Glycitein, Napabucasin, 3-(5-(4-(cyclopentyloxy)-2-hydroxybenzoyl)-2-((3-hydroxybenzo[d]isoxazol-6-yl) meth-oxy) phenyl) propanoic acid, and Tanshinone IIA. The chemical structures of these compounds, and the hydrogen bonds surrounding the carbon compound, are illustrated in Figure 5.

### 2.5. Verification by Molecular Docking

**Figure 6:**
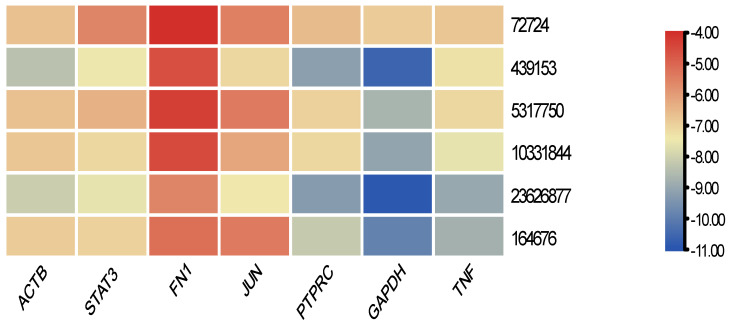
Heat map displaying the binding affinities (ΔG) of drugs against a panel of genes. The *x*-axis shows the genes analyzed, while the *y*-axis displays the drug molecules tested. The color scale represents the affinity range from −4.00 to −11.00 kcal/mol, where blue indicates higher affinity (more negative ΔG values), and red indicates lower affinity (less negative ΔG values). The binding affinities were calculated using molecular docking. (72724: Alantolactone, 439153: 1,4-dihydronicotinamide adenine dinucleotide, 5317750: Glycitein, 10331844: Napabucasin, 23626877: 3-(5-(4-(Cyclopentyloxy)-2-hydroxybenzoyl)-2-((3-hydroxybenzo[d]isoxazol-6-yl) methoxy) phenyl) propanoic acid, 164676: Tanshinone IIA).

**Figure 7:**
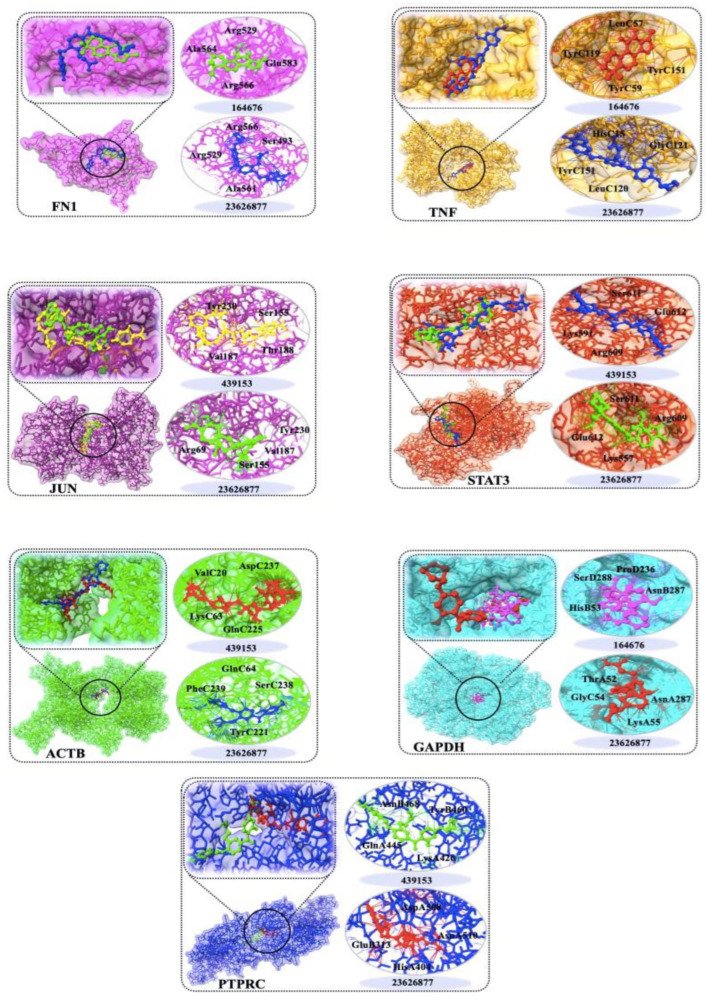
Docking of small molecule drugs with targets. The active site of protein is shown. Hydrogen bonds and van der Waals interactions between the drugs and protein X are depicted by orange and green dotted lines, respectively. Several important binding interactions are labeled. The molecular docking analysis was performed using AutoDock Vina.

Using PyRx 0.8, the screened small-molecule drugs were docked with seven core targets (ACTB, STAT3, FN1, JUN, PTPRC, GAPDH, and TNF). A binding energy lower than 0 suggests that the ligand and receptor bind spontaneously. The possibility of a higher activity was determined by the stability and binding energy of the conformation. The lowest binding energy was usually less than −11 kcal mole^−1^, showing that the target protein had a high affinity for the active component. The summary of binding energies is shown in Figure 6. A small molecule therapeutic docking target was developed. The bonding of a drug molecule and its interaction with identified genes is shown in Figure 7. For an example, Tanshionone IIA exerts its biological activity by attaching to, and creating, hydrogen bonds with four amino acids near the active site, as represented by the dotted line.

## 3. Discussion

Traditional gene expression analysis poses a challenge considering the biological heterogeneity and technical biases of sequencing platforms [23]. In this study, we utilized methods to eliminate these limitations through a robust normalization and scaling based on the relative ranks of gene expression levels, as proposed by several previous studies [24,25]. In this study, we identified 1481 DEGs, including 381 upregulated and 1100 downregulated genes. Further analysis narrowed down our search to seven hub genes: FN1, TNF, JUN, STAT3, ACTB, GAPDH, and PTPRC. Through CMap and molecular docking, we found that 3-(5-(4-(cyclopentyloxy)-2-hydroxybenzoyl)-2-((3-hydroxybenzo[d]isoxazol-6-yl) methoxy) phenyl) propanoic acid (PubChem ID: 23626877) could be used to target all of the postulated genes to reverse BRONJ.

All hub genes were directly or indirectly involved in bone metabolism. The FN1 gene provides instructions for producing fibronectin-1 protein [26]. FN1 has been shown to mediate chondrocyte adhesion [27]. Upregulation of FN1 mediates fracture healing by activating the TGF-B/PI3K/Akt signaling pathway [28]. TNF is a protein-coding gene that is associated with malaria and asthma [29]. Additionally, TNF plays an important role in skeletal system-induced inflammatory processes [30]. Jun encodes proteins via GTPase activator activity. In several studies, Jun has been postulated to cause bone development [31,32,33]. ACTB provides instructions for the expression of B-actin. In a study on postmenopausal osteoporosis subjects, it was shown that ACTB was aberrated by being an unsuitable house-keeping gene for expression assays [34]. GAPDH is a moonlighting protein that serves as a glycolytic enzyme and an uracil DNA glycosylase [35]. In a study conducted to investigate the possible effects of BP on GAPDH mRNA expression, GAPDH expression decreased, similar to our findings [36]. PTPRC is a tyrosine phosphatase (PTP) protein. PTPRC is also known as the CD45 antigen or leukocyte common antigen (LCA) [37].

**Figure 8:**
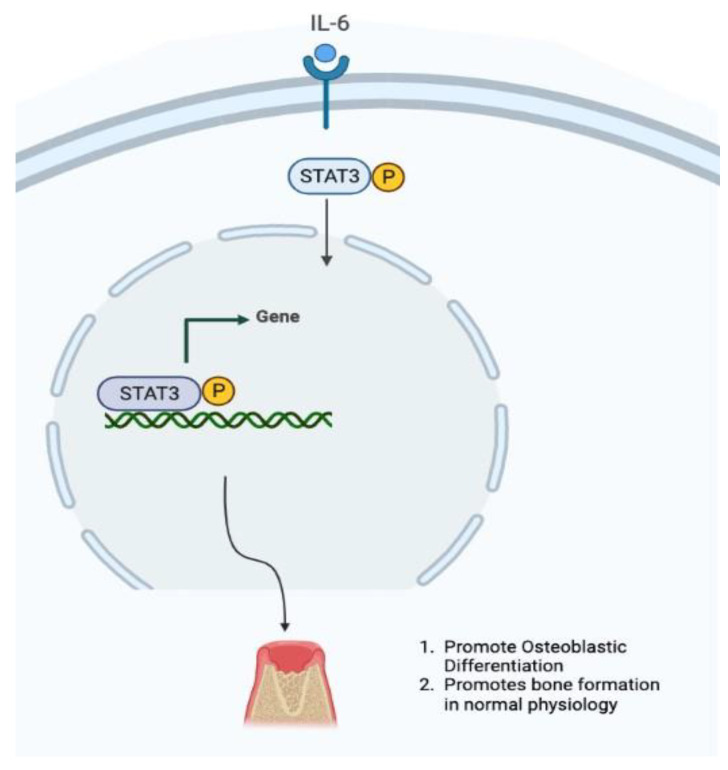
The IL-6 activated by inflammatory triggers initiates the pathway by phosphorylation of STAT3.

Based on our scoping review of the genes studied in BRONJ, STAT3 is postulated to be activated subsequent to the immune response. Treatment with BP elevates IL-6 and IL-36a levels, which then activates the STAT3 pathway [38]. The molecular function of this pathway is illustrated in Figure 8. Several studies have shown the differential pathways of nitrogenous versus non-nitrogenous BP on STAT3. Disruption of this pathway may effectively control RANKL expression.

3-{5-[4-(cyclopentyloxy)-2-hydroxybenzoyl]-2-[(3-hydroxy-1,2-benzoxazol-6-yl) methoxy] phenyl} propanoic acid is an organic compound that is classified as a benzophenone, which has a ketone attached to two phenyl groups. Despite being a relatively unknown compound, it has been detected in human blood, indicating that it is not a naturally occurring metabolite, and is only present in people who have been exposed to it or its derivatives [39]. This compound is considered part of an individual’s exposome, which encompasses all the environmental and occupational exposures that affect their health throughout their lifetime, starting before birth [39]. This study provides a basis for the further exploration and validation of 3-{5-[4-(cyclopentyloxy)-2-hydroxybenzoyl]-2-[(3-hydroxy-1,2-benzoxazol-6-yl) methoxy] phenyl} propanoic acid for therapeutic purposes.

This study lends itself to several limitations. Although bioinformatics data mining can provide valuable information, available datasets, particularly those related to BRONJ, are limited. As such, we utilized a single center study. Additionally, a single chip analysis should be interpreted with caution as it may account for limited coverage, technical variability, and cross hybridization, making it less feasible to arrive at a plausible conclusion.

## 4. Materials and Methods

### 4.1. Flowchart of Study

**Figure 9:**
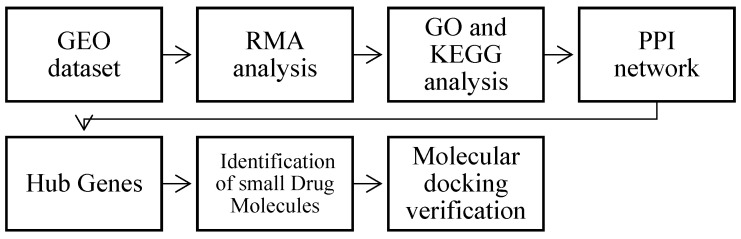
General overview of the research pipeline.

Figure 9 illustrates the pipeline of this study. Firstly, the raw data from a gene expression experiment or a protein study was collected and compiled in a geo dataset. Then, the data was preprocessed and analyzed using statistical methods, such as RMA analysis, to normalize and filter the data. Furthermore, gene ontology (GO) and a Kyoto Encyclopedia of Genes and Genomes (KEGG) analysis were performed to identify the biological processes, molecular functions, and pathways that were significantly enriched in the dataset. Subsequently, a protein–protein interaction (PPI) network was constructed to visualize the interactions between different proteins and to identify the highly connected hub genes that play a crucial role in the biological system being studied. Once the hub genes were identified, small drug molecules were screened and identified using virtual screening methods to target the protein products of these hub genes. A molecular docking verification was then performed to predict the binding affinity and the stability of the drug–protein complex.

### 4.2. Differential Expression Analysis

The expression profile for BRONJ was obtained from the Gene Expression Omnibus (GEO) database [(https://www.ncbi.nlm.nih.gov/, accessed on 28 November 2022)]. The search strategy [“bisphosphonate related osteonecrosis of the jaw” (MeSH Terms) OR “bisphosphonate related osteonecrosis of the jaw” (All fields)] and [“Home sapiens” (Organism) and “Expression profiling” (Filter)] were used. The data contained 11 multiple myeloma (MM) patients with BRONJ, against 10 age-matched MM patients without BRONJ. Further details are provided in Table 2. The ‘limma’ package was used to normalize the data in R using the RMA method (version 4.2.2, http://www.R-project.org/, accessed on 28 November 2022). Using the R package ‘limma,’ a linear model was used to assess differential expression. The cut-off parameters for determining DEGs were [log2 fold change (FC)] > 1 and *p*-value *p* < 0.05.

### 4.3. Gene Ontology and Pathway Analysis

GO enrichment and KEGG pathway analyses were performed using a freely available Database for Annotation, Visualization, and Integrated Discovery (DAVID) (DAVID 6.8, http://david.ncifcrf.gov/, accessed on 28 November 2022). GO annotates gene products and functions into three categories: biological processes (BP), molecular functions (MF), and cellular components (CC), allowing us to compare and analyze genes across control versus BRONJ subjects. A *p*-value of 0.05 was used as the cutoff criterion.

### 4.4. Protein–Protein Interaction (PPI) Network Analysis

The STRING online database was used to construct a functional PPI network for the identified DEGs (https://string-db.org/, accessed on 28 November 2022). The cut-off point was set to a credibility score higher than 0.4. PPI visualization was performed using the Cytoscape software.

### 4.5. Hub Gene Mapping

The Cytoscape cytoHubba plugin was used to select hubs from the DEGs. (https://apps.cytoscape.org/apps/cytohubba, accessed on 28 November 2022). Cytoscape is an open-source software platform used for visualizing and analyzing molecular interaction networks. CytoHubba is a plugin for Cytoscape that provides various network analysis algorithms to identify important nodes or subnetworks within a given network. Several topological algorithms were used to identify interactome networks in this plug-in. The following metrics were used: density of the maximum neighborhood component (MNC), maximal clique centrality (MCC), density of the maximum neighborhood component (DMNC), closeness, betweenness, and degree. Venn diagrams were used to locate the shared genes.

### 4.6. Identification of Small Molecule drug Candidates

To assess the potential effectiveness of the medication treatment for BRONJ, we measured the binding energy between the small-molecule medications predicted in CMap and the possible target proteins of BRONJ. The crystal structures of the principle targets were obtained from the RCSB Protein Data Bank (PDB, http://www.rcsb.org, accessed on 28 November 2022), whereas the mol2 file formats of the compounds were retrieved from the PubChem database. Target proteins were extracted from PyMOL 2.3.2, and their ligands were stored in PDB format. The target protein was then hydrogenated, and its charge was calculated and stored in PDBQT format using AutoDock Tools 1.5.6 software. Grid box data for the protein of interest were obtained, and molecular docking was performed using AutoDock Vina 1.1.2. The results of molecular docking were visualized using PyRx software. PyRx 0.8 is a useful tool for computational drug discovery and design, used to perform virtual screening experiments by docking small molecule ligands to protein targets.

## 5. Conclusions

There is currently no known biomarker for BRONJ. The present study used bioinformatics to analyze the gene chip data of MM patients with BRONJ. The resulting findings allowed us to identify potential molecular biomarkers for BRONJ. Apart from that, we were also able to identify potential therapeutic targets for BRONJ. All identified hub genes were either directly or indirectly related to bone metabolism, which could potentially be aberrated in disease, and hence a factor in the development of BRONJ. The present results are based only on bioinformatics analysis, and further validation is required to verify this finding. Further efforts in utilizing microarray studies may benefit efficient biomarker development.

## Figures and Tables

**Table 1:** Summary of studies that have used GSE7116 dataset.

Study	Study Area	Study Objectives	Normalization	Screening of Small Drug	Molecular Docking	Reference
Kim et al., 2017	BRONJ	To identify combined biomarkers associated with BRONJ	No preprocessing of data	NO	NO	[13]
Dong Leng et al., 2018	Multiple Myeloma (MM)	To investigate transcriptional changes of CRISP3 for MM marker	CONOR_1.0.1	NO	NO	[16]
Jiangnan Sun et al., 2015	BRONJ	To explore molecular mechanisms associated with BRONJ in patients with MM	RMA	NO	NO	[17]
Juncheng et al., 2020	Immune Responses in BRONJ	To investigate the immune cellular and genomic profiles of BRONJ and excavate potential small molecule drug	Not Mentioned but screened only immune related genes	CMap	NO	[18]
Huanzhi Ma et al., 2021	Osteonecrosis	To investigate the DEGs of normal vs. osteonecrosis patients (GSE74089, GSE7116, GSE123568)	lmFit and eBayes	NO	NO	[19]
Jinpeng Zhuang et al., 2022	BRONJ	To identify drugs that potentially modulate the risk of BRONJ in cancer	GEO2R	DGIdb	NO	[14]
Our Present Study	BRONJ	To identify biomarkers and small drug molecules related to BRONJ	RMA	CMap	YES	

**Table 2:** Details of GEO BRONJ data.

Reference	GEO Number	Platform	BRONJ (*n*)	Control (*n*)	Region	Reference
Raje et al. (2008)	GSE7116	GPL570	11	10	USA	[40]

## Data Availability

All data can be easily obtained and linked in the respective sections.

## Supplementary Materials

The supporting information can be downloaded at: https://www.mdpi.com/article/10.3390/ijms24108635/s1.
